# Supplementary figures and images for: Colony-stimulating factor 3 signaling in colon and rectal cancers: Immune response and CMS classification in TCGA data
Source: PLoS One. 2021 Feb 19;16(2):e0247233. doi: 10.1371/journal.pone.0247233 (PMC7895368; doi:10.1371/journal.pone.0247233)

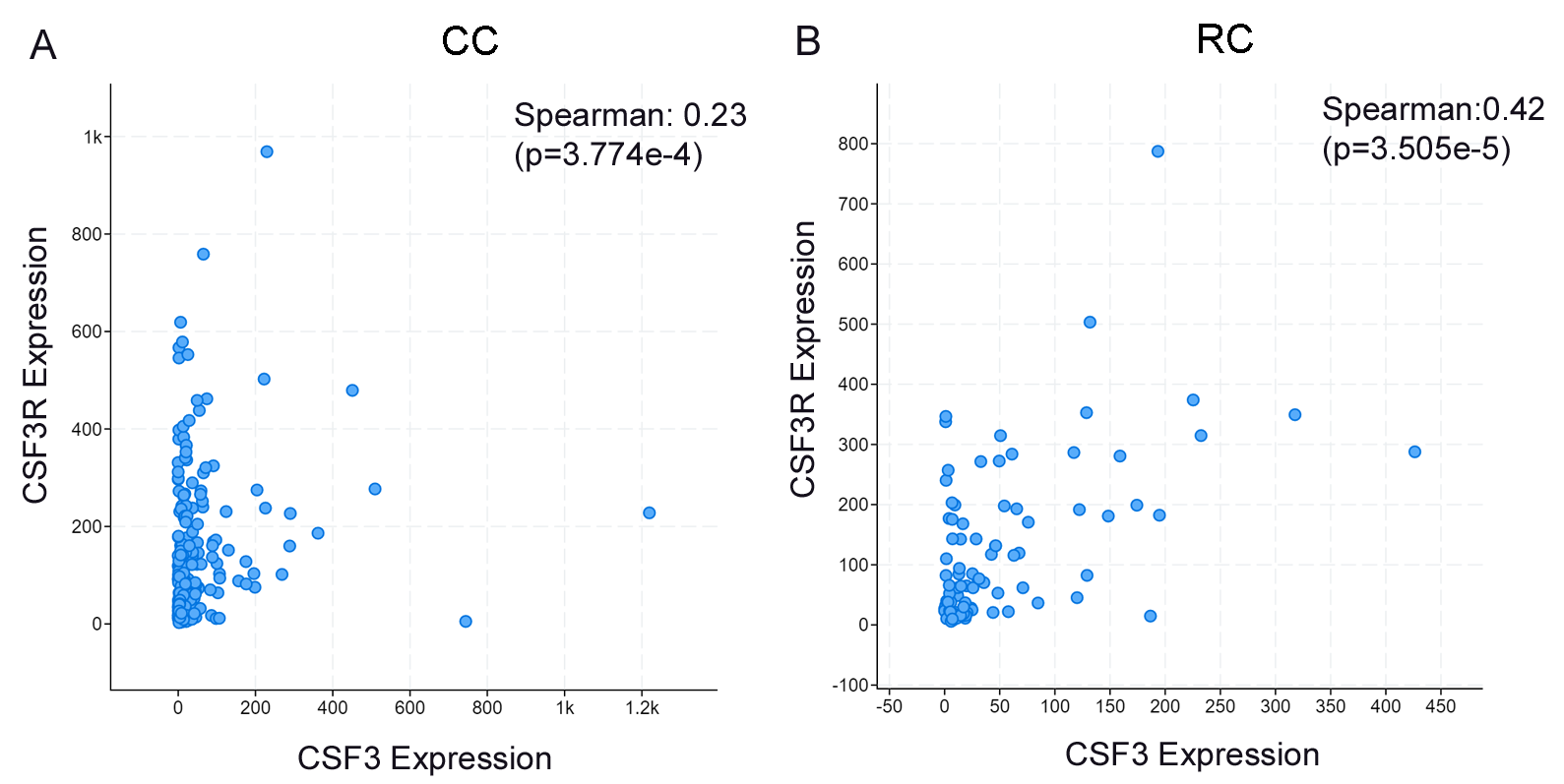

Supplement: S1 Fig — Scatterplots depicting Spearman’s correlation of TCGA Firehose Legacy dataset between CSF3R and CSF3 in CC (A) and RC (B). (TIF) [file pone.0247233.s001.tif]

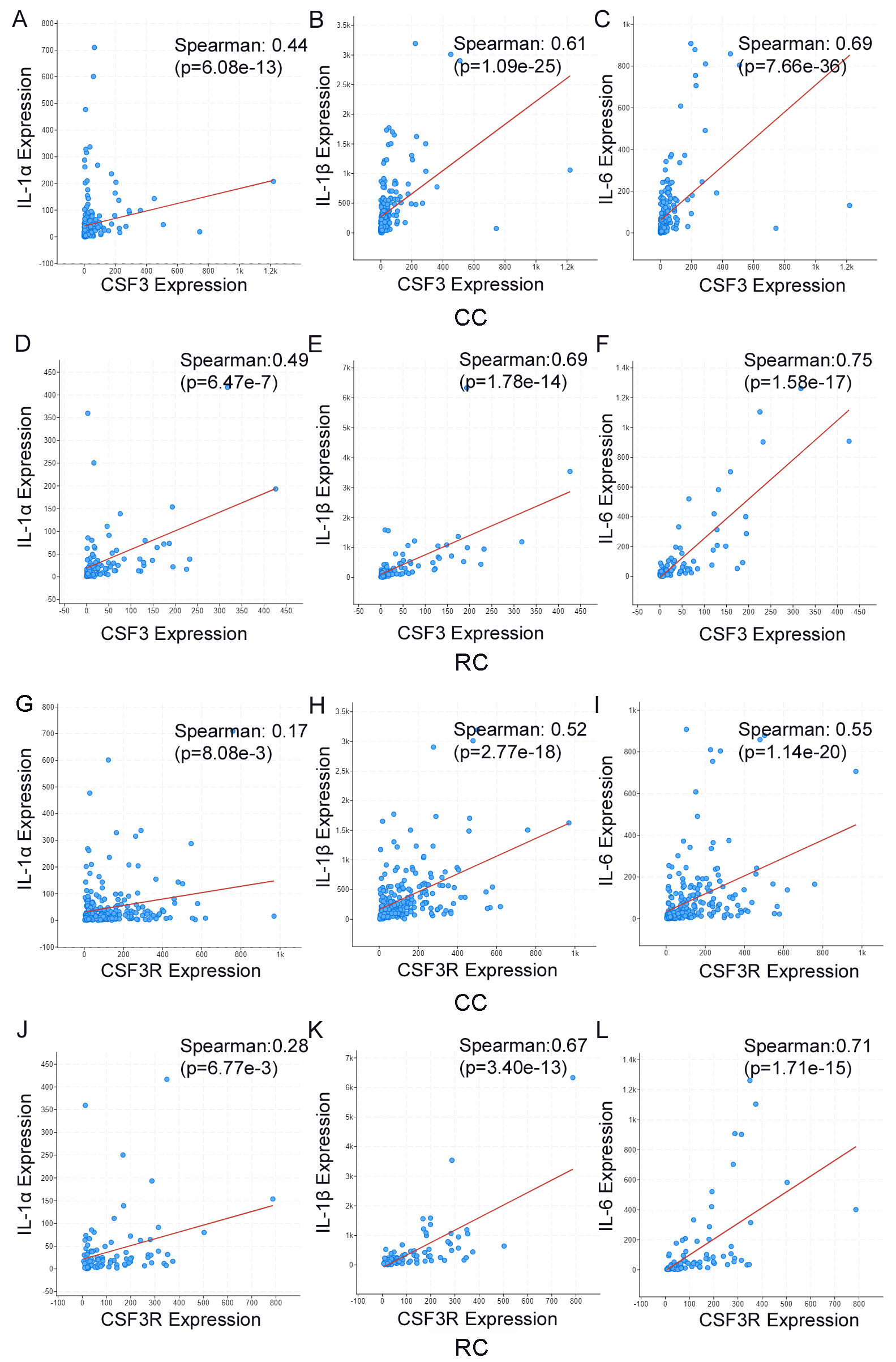

Supplement: S2 Fig — Scatterplots depicting Spearman’s correlation of TCGA Firehose Legacy dataset between IL-1α, IL-1β, or IL-6 and CSF3 in CC (A-C) and RC (D-F); and IL-1α, IL-1β, or IL-6 and CSF3R in CC (G-I) and RC (J-L). Red line indicates linear regression. (TIF) [file pone.0247233.s002.tif]

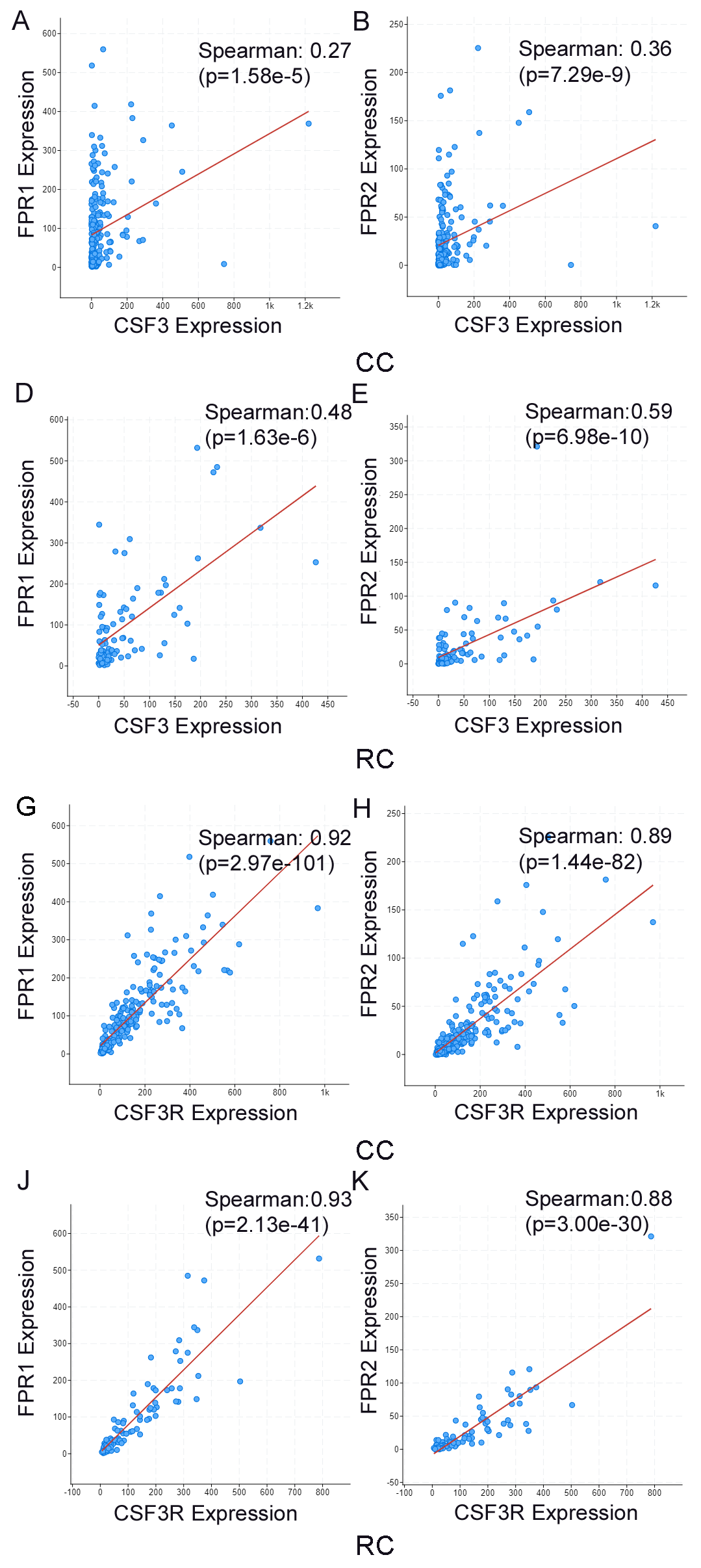

Supplement: S3 Fig — Scatterplots depicting Spearman’s correlation of TCGA Firehose Legacy dataset between FPR1 or FPR2 and CSF3 in CC (A, B) and RC (C, D); and FPR1 or FPR2 and CSF3R in CC (E, F) and RC (G, H). Red line indicates linear regression. (TIF) [file pone.0247233.s003.tif]

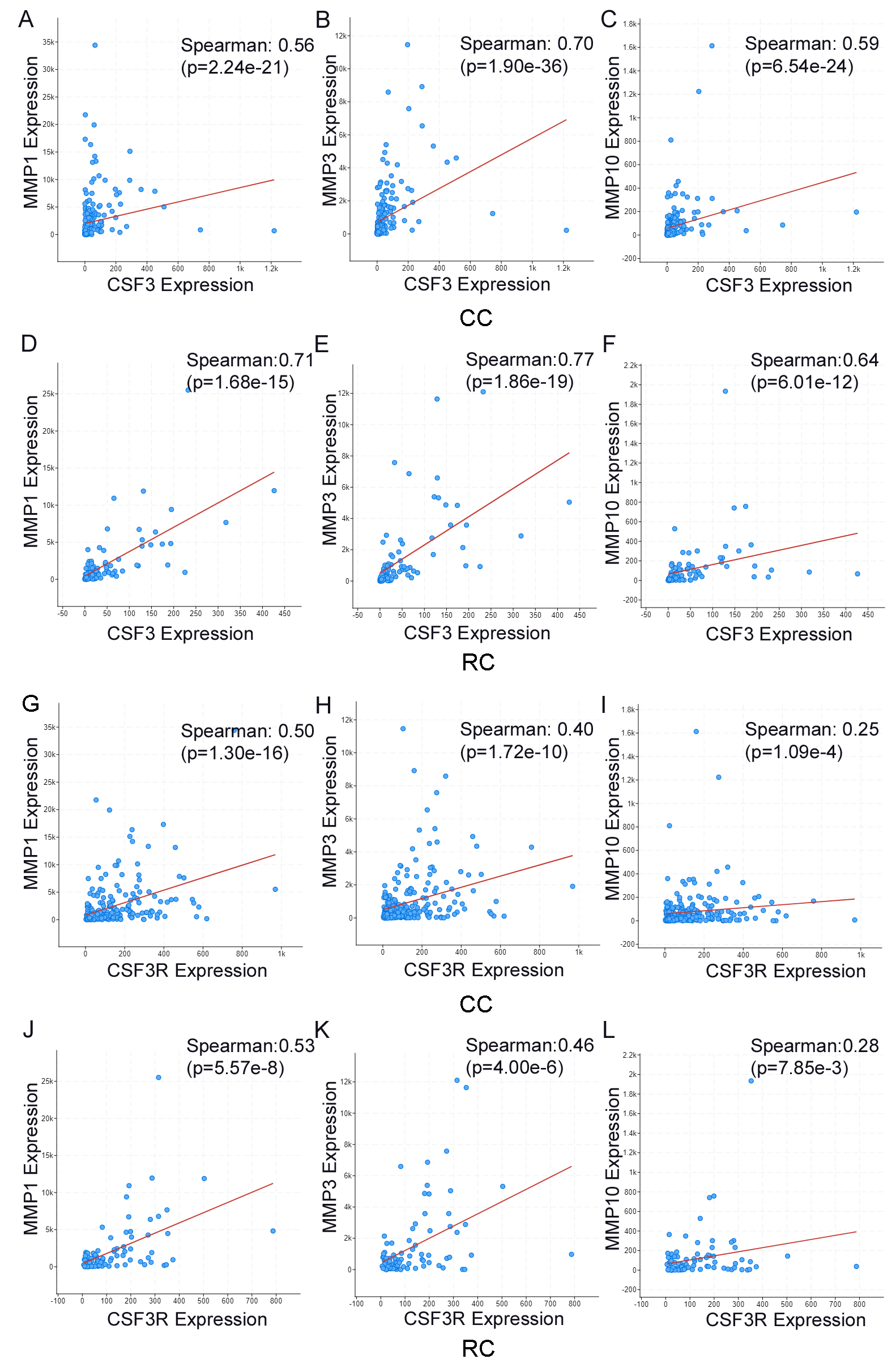

Supplement: S4 Fig — Scatterplots depicting Spearman’s correlation of TCGA Firehose Legacy dataset between MMP1, MMP3 or MMP10 and CSF3 in CC (A-C) and RC (D-F); and MMP1, MMP3 or MMP10 and CSF3R in CC (G-I) and RC (J-L). Red line indicates linear regression. (TIF) [file pone.0247233.s004.tif]
