# Supplementary material for: Colony-stimulating factor 3 signaling in colon and rectal cancers: Immune response and CMS classification in TCGA data
Source: PLoS One. 2021 Feb 19;16(2):e0247233. doi: 10.1371/journal.pone.0247233 (PMC7895368; doi:10.1371/journal.pone.0247233)
Supplement: Attachment — Submitted filename: Response to Reviewer Saunders CSF3 TCGA.pdf [file pone.0247233.s006.pdf]

The University of Oklahoma
Health Sciences Center

Katherine Morris, MD, FACS, FRCP Edin
Associate Professor
G. Rainey Williams Chair for Surgical Research
Univ. of Oklahoma Health Sciences Center
Dept. of Surgery
College of Medicine Building,
PO Box 26901, Suite 9000
Oklahoma City, OK. 73126

Wednesday, January 27, 2021
Re: PLOS ONE Paper PONE-D-20-30842

Dear Dr. Pizzo,

We thank the editor and reviewer for their thoughtful review of our manuscript titled "Colony-stimulating factor 3 signaling in colon and rectal cancers: Immune response and CMS classification in TCGA data." We appreciate the insightful comments as well as the chance to resubmit the work. Below is an itemized response to the referee's comments.

Journal Requirements
We have revised the manuscript to meet PLOS ONE's style requirements, including those for file naming.
Comment: TCGA is listed in the introduction line 72 for the first time without being defined. Please define for the reader. The same is true of ESTIMATE.
Response: We have defined these in the introduction (line 73) and expanded upon the definition of ESTIMATE in the methods section (line 76).

Comment: I find the use of nonconventional abbreviations for colon and rectal cancer (COAD and READ) to be distracting. Why not CC and RC?
Response: We have changed the abbreviations to CC and RC for colon and rectal cancer.

Comment: It seems as though you are implying that because colon and rectal cancers are treated somewhat differently they must be molecularly distinct. If this is indeed what you are implying it is an erroneous statement as rectal cancers are treated with radiation purely because of their location in the body. Likely colon cancer would have a similar response to irradiation, but this is not feasible due to its proximity to small bowel and the risk of radiation injury.
Response: We appreciate the reviewer's comment and have changed the text in the Methods (line 93) and the Discussion (line 411) to reflect that we analyzed colon and rectal cancers separately due to concerns about the potential for significant changes in the transcriptomics due to preoperative chemoradiation therapy.

Comment: TCGA should be described in your materials and methods section.
Response: We are grateful for the attention that was given to the materials and methods section. We have highlighted TCGA as a stand-alone material (line 87) to address this.

Comment: Presumably dysregulation of the CF3 pathway will occur at different points in the mutational cascade for different tumors. Do you think that changes in the regulation of CSF3 in this cohort are the result of a primary mutation on the CSF3 and CSF3R genes or is this secondary to other genes/proteins effects on the regulation of CSF3 and CSF3R?

Post Office Box 26901, Andrews Academic Tower, Suite 9000, Oklahoma City, Oklahoma 73126-0901
PHONE: (405) 271-7912 FAX: (405) 271-3919

The University of Oklahoma
Health Sciences Center

Response: Thank you for drawing our attention to this excellent point. In TCGA data, the rate of mutations within the CSF3 or CSF3R genes in CRC samples is less than 2%. Based on that low rate of mutations in these genes, we believe observed associations are likely secondary to the influence of other tumor mutations, as well as the cytokine milieu, immune infiltrate, and stromal phenotype. We have published data from mouse models suggesting that in inflammation-associated cancers, CSF3 signaling is critical to tumor development at early stages, and increases during tumor development with no mutations in CSF3 or CSF3R. We have updated the Discussion (line 397) to include the frequency of mutations of CSF3 and CSF3R and reflect some of the other considerations.

Comment: Are you able to determine at what point in tumorigenesis CSF3 becomes a key player in further tumor progression based on the ontological markers you have identified?
Response: We thank the reviewer for this insightful comment. The correlations we observe between Wnt signaling and the ERK/MAPK activity with CSF3 and CSF3R leads us to believe that increase in CSF3 signaling occurs early in CRC development. We have added this to the Discussion (line 380).

Comment: Your results section spends a significant amount of time explaining techniques like ESTIMATE and concepts like CMS1-4. These should be explained prior to the results section, either in the introduction or the methods and results should only be included in the results section.
Response: We have adjusted the manuscript to include these descriptions in the introduction and methods section and appreciate the reviewer's help in increasing the clarity of the work.

We hope these alterations and responses have strengthened the manuscript and appreciate the chance to revise and resubmit this work.

Sincerely,

Katherine Morris MD, FACS

Post Office Box 26901, Andrews Academic Tower, Suite 9000, Oklahoma City, Oklahoma 73126-0901
PHONE: (405) 271-7912 FAX: (405) 271-3919
